# Elevated gamma glutamyl transferase levels are associated with the location of acute pulmonary embolism. Cross-sectional evaluation in hospital setting: Níveis elevados de gama glutamil transferase estão associados com a localização de embolia pulmonar aguda. Estudo transversal em ambiente hospitalar

**DOI:** 10.1590/1516-3180.2015.00131806

**Published:** 2015-12-08

**Authors:** Ozge Korkmaz, Hasan Yucel, Ali Zorlu, Ocal Berkan, Hakki Kaya, Sebahattin Goksel, Osman Beton, Mehmet Birhan Yilmaz

**Affiliations:** I MD. Associate Professor, Department of Cardiovascular Surgery, Cumhuriyet University Medical Faculty, Sivas, Turkey.; II MD. Associate Professor, Department of Cardiology, Cumhuriyet University Medical Faculty, Sivas, Turkey.; III MD. Professor and Head of Department of Cardiovascular Surgery, Cumhuriyet University Medical Faculty, Sivas, Turkey.; IV MD. Professor and Head of Department of Cardiology, Cumhuriyet University Medical Faculty, Sivas, Turkey.

**Keywords:** Gamma-glutamyltransferase, Pulmonary embolism, Biological markers, Pulmonary artery, Multidetector computed tomography

## Abstract

**CONTEXT AND OBJECTIVE:**

The location of embolism is associated with clinical findings and disease severity in cases of acute pulmonary embolism. The level of gamma-glutamyl transferase increases under oxidative stress-related conditions. In this study, we investigated whether gamma-glutamyl transferase levels could predict the location of pulmonary embolism.

**DESIGN AND SETTING:**

Hospital-based cross-sectional study at Cumhuriyet University, Sivas, Turkey.

**METHODS:**

: 120 patients who were diagnosed with acute pulmonary embolism through computed tomography-assisted pulmonary angiography were evaluated. They were divided into two main groups (proximally and distally located), and subsequently into subgroups according to thrombus localization as follows: first group (thrombus in main pulmonary artery; n = 9); second group (thrombus in main pulmonary artery branches; n = 71); third group (thrombus in pulmonary artery segmental branches; n = 34); and fourth group (thrombus in pulmonary artery subsegmental branches; n = 8).

**RESULTS:**

: Gamma-glutamyl transferase levels on admission, heart rate, oxygen saturation, right ventricular dilatation/hypokinesia, pulmonary artery systolic pressure and cardiopulmonary resuscitation requirement showed prognostic significance in univariate analysis. The multivariate logistic regression model showed that gamma-glutamyl transferase level on admission (odds ratio, OR = 1.044; 95% confidence interval, CI: 1.011-1.079; P = 0.009) and pulmonary artery systolic pressure (OR = 1.063; 95% CI: 1.005-1.124; P = 0.033) remained independently associated with proximally localized thrombus in pulmonary artery.

**CONCLUSIONS:**

: The findings revealed a significant association between increased existing embolism load in the pulmonary artery and increased serum gamma-glutamyl transferase levels.

## RESUMO

**CONTEXTO E OBJETIVO:**

: A localização da embolia está associada com os resultados clínicos e a gravidade da doença do embolismo pulmonar agudo (EPA). O nível de gama-glutamil transferase (GGT) aumenta em condições relacionadas com estresse oxidativo. Investigou-se se os níveis de GGT podem prever a localização do EPA.

**TIPO DE ESTUDO E LOCAL:**

: Estudo observacional transversal na Universidade Cumhuriyet, Sivas, Turquia.

**MÉTODOS:**

: Avaliamos 120 pacientes diagnosticados com EPA após a realização de angiografia pulmonar assistida por tomografia computadorizada. Eles foram divididos em dois grupos principais (localização proximal e distal) e depois em subgrupos de acordo com a localização do trombo da seguinte forma: primeiro grupo (trombo na artéria pulmonar [AP] principal, n = 9); segundo (trombo no ramo da AP principal; n = 71); terceiro grupo (trombo na segmentar da AP; n = 34); quarto grupo (trombo na subsegmentar da AP; n = 8).

**RESULTADOS:**

: Na análise univariada, os níveis de GGT tiveram significado prognóstico em relação à admissão, pulsação arterial, saturação de oxigênio, dilatação do ventrículo direito/hipocinesia, pressão sistólica da artéria pulmonar (PSAP) e necessidade de ressuscitação cardiopulmonar. O modelo de regressão logística multivariada demonstrou que o nível de GGT na admissão (razão de possibilidades, OR: 1,044; 95% intervalo de confiança, CI: 1,011-1,079; P = 0,009) e PSAP (OR: 1,063, 95% CI: 1,005-1,124; P = 0,033) permaneceram independentemente associados com trombo localizado proximalmente na AP.

**CONCLUSÕES:**

: Os resultados demonstraram associação significativa entre aumento da carga existente de embolia da AP e aumento dos níveis séricos da GGT.

## INTRODUCTION

Acute pulmonary embolism (APE) is a common disease resulting from acute full or partial obstruction of the pulmonary artery bed and can lead to disability or mortality.^1^ ^2^ In the majority of APE patients, mortality occurs as a result of hemodynamic failure and, for this reason, mortality rates are significantly high.^3^ ^4^ The general rule for treatment is to diagnose patients as early as possible, so as to determine the risk factors and begin treatment.^1^ Different clinical evaluations, radiological examinations (including echocardiography) and blood biomarkers are analyzed for diagnosis and risk classification. The location of the embolism is associated with clinical findings and disease severity.^5^ Cardiac biomarkers, including troponin and natriuretic peptide, are used as mortality predictors for APE.^1^ ^6^ Although some studies have indicated a correlation between the location of the embolism and blood D-dimer and creatinine levels, others indicate the opposite, i.e. suggesting that there is no correlation.^7^ ^8^

Gamma-glutamyl transferase (GGT) is a membrane protein that functions in the extracellular catabolism of glutathione. It is mostly synthesized in the liver, but also in various other organs. GGT levels become increased especially in cases of hepatobiliary dysfunction and alcohol consumption, and recent studies have shown that GGT levels increase under oxidative stress-related conditions, including inflammation, carcinogenesis, ageing, atherosclerosis and reperfusion injury.^9^ ^11^ The GGT level is independently associated with the prognosis in certain cardiopulmonary diseases, especially acute coronary syndrome, acute myocardial infarction and heart failure, and has also been described as a predictor of early mortality in cases of acute pulmonary embolism.^12^ ^14^

## OBJECTIVES

The objective of this study was to investigate the association between serum GGT levels and the location of APE.

## METHODS

The current study is a subgroup analysis on a study previously published.^14^ In this study, a total of 192 consecutive patients who were admitted to the emergency unit and then hospitalized with suspected APE were considered for enrolment between January 2009 and July 2011. Excluded from the study were 15 patients with cholecystitis, seven patients with chronic liver disease, 4 patients who were receiving dialysis treatment for chronic renal failure, 5 patients with a previous diagnosis of malignancy, 2 patients who were chronic consumers of alcohol, 20 patients whose serum GGT had not been measured and 12 patients in whom the diagnosis of APE was ruled out through scintigraphy and/or tomography (alternative diagnoses were confirmed). Also excluded from the current study were patients who were diagnosed with APE through imaging methods other than computed tomography-assisted pulmonary angiography (ventilation/perfusion scintigraphy, n = 2) and those with incomplete medical records (n = 3). In the end, evaluations were made on 122 patients (female/male: 63/59) who were admitted to the emergency service, and who were diagnosed with APE following computed tomography-assisted pulmonary angiography. 

After the results from contrast spiral computed tomography had been obtained, the patients were divided into two main groups (proximally and distally located), and these two main groups were then divided into subgroups according to thrombus localization as follows: first group (thrombus in main pulmonary artery; n = 9); second group (thrombus in main pulmonary artery branches; n = 71); third group (thrombus in pulmonary artery segmental branches; n = 34); and fourth group (thrombus in pulmonary artery subsegmental branches; n = 8). Embolisms localized in the pulmonary trunk, main pulmonary arteries and lower arteries were classified as "proximally located", while lobular-segmental-located and subsegmental embolisms were classified as "distally located".

All the patients were evaluated in accordance with the clinical, laboratory and radiological findings regarding the location of APE. The following information was obtained from the medical records, and was evaluated retrospectively in detail: identity information, demographic data, symptoms, risk factors, comorbidities (history of coronary artery disease, diabetes mellitus, hypertension and chronic obstructive pulmonary disease), physical examinations, laboratory findings, 12-lead electrocardiography, echocardiography and computed tomography-assisted pulmonary angiography findings. 

Vital signs and blood samples were obtained within 30 minutes of admission to the emergency unit. GGT activity was measured by means of a Beckman Coulter Synchron LX20 (Brea, CA, USA) autoanalyzer, using original kits. The laboratory reference limits differ significantly according to sex, and were set at 9 U/l to 35 U/l for women and 9 U/l to 40 U/l for men, in accordance with the test kit specifications. 

Hypertension was defined as having a blood pressure of 140/90 mmHg or greater on more than two occasions during measurements, or undergoing antihypertensive treatment. Diabetes mellitus was defined as having a fasting blood glucose level of 126 mg/dl or greater, or undergoing anti-diabetic treatment. The presence of coronary artery disease was defined as having abnormal stress test results with evidence of ischemia, or a documented coronary stenosis of more than 50% on a coronary angiogram, or having a clinical history of coronary artery disease. Rhythm and electrocardiographic findings relating to right ventricle loading, including S1Q3T3, right bundle-branch block pattern and T-wave changes in the right precordial derivations, were evaluated. Furthermore, the duration of hospitalization after the APE diagnosis was recorded; and pulmonary embolism-associated early mortality events were recorded as pulmonary embolism-dependent in-hospital mortality. 

Echocardiography was carried out within the first 24 hours of admission to the emergency room. All examinations were evaluated via the Vivid 7 system (GE Healthcare, Wauwatosa, WI, USA) in all participating centers using a 2.5 to 5-MHz probe. The modified Simpson method was used to calculate ejection fractions, with chamber sizes defined in line with recent guidelines.^15^ Right ventricular dysfunction was defined as dilatation of the right ventricle (right ventricle dimension > 3.4 cm at basal plane or > 3.8 cm at the midplane), combined with the presence of McConnell's sign.^15^ ^16^ Severe tricuspid regurgitation was defined using color flow jet Doppler signal intensity, in combination with vena contracta width, in accordance with recommendations.^17^ Pulmonary artery systolic pressure was calculated as previously described.^18^

First posteroanterior lung graphs were produced. Patients with suspected pulmonary embolism were evaluated in detail within the first 24 hours in the radiology clinic, through contrast spiral computerized tomography imaging (imaging performed with 120 kV, 210-250 mAs, slice thickness of 3 mm, table speed of 5 mm/s, reconstruction index of 2 mm, pitch of 2 and 120 ml of intravenous contrast dye). The pulmonary artery, its branches and the lung parenchyma were examined in detail. 

Continuous variables that did not have normal distribution were expressed as mean ± standard deviation (SD) or as the interquartile range, while the categorical variables were expressed as percentages. A receiver operator characteristic curve analysis was carried out to identify the optimal cutoff point for GGT (at which sensitivity and specificity would be maximum), in order to predict the proximal localization of the arteries. The area under the curve (AUC) was calculated as a measure of the accuracy of the tests. The researchers compared the AUC using a Z test. Patients with APE were classified into two main groups: proximal localization of the artery (Group I) and distal localization of the artery (Group II). Comparisons between groups of patients were made using a χ^2^ test for categorical variables, an independent-sample t test for normally distributed continuous variables and a Mann-Whitney U test when the distribution was skewed. An evaluation of the correlation between the variables was made using either Pearson's or Spearman's correlation test. We conducted univariate analysis to quantify the associations of variables with proximally localized thrombus in pulmonary artery. The variables found to be statistically significant in the univariate analysis and other potential confounders were used in a multiple logistic regression model using the forward stepwise method in order to determine the independent prognostic factors for proximal artery localization. In the logistic regression analyses, GGT was evaluated as a continuous variable. All statistical analyses were carried out using the SPSS software version 14.0 (SPSS Inc., Chicago, IL, USA), and P values < 0.05 were considered to be statistically significant.

## RESULTS

Enrolled in the study were 122 consecutive patients with confirmed APE, with a mean age of 64 ± 13 years (52% female, 48% male). 

**Figure 1.**
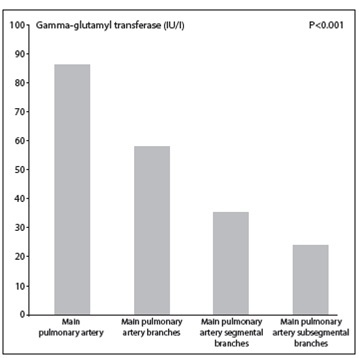
Comparison of gamma-glutamyl transferase (GGT) levels between four groups.

The comparisons between the four subgroups of patients with APE, along with their baseline characteristics and hemodynamic, electrocardiographic, echocardiographic and laboratory findings, are summarized in Table 1. The baseline characteristics and all previously mentioned electrocardiographic parameters were similar in all groups. Most of the transthoracic echocardiograms were examined within the first 24 hours of emergency unit admission (upon admission for most of the patients), while echocardiographic examinations with extended deadlines (n = 19) were not considered in this study due to the potential for altered values. No significant differences were identified between the groups regarding left ventricle ejection fraction levels; however, pulmonary artery systolic pressure levels (an indicator of right ventricle load) and right ventricular dilatation rates in the right heart cavities increased as the severity of pulmonary artery involvement increased. When the patients' hemodynamic parameters were evaluated, heart rates were found to increase with increasing pulmonary artery involvement, whereas oxygen saturation decreased. No significant difference was observed between systolic and diastolic blood pressures. An evaluation of the laboratory parameters revealed that GGT levels increased and albumin levels decreased as the severity of pulmonary artery involvement increased (Figure 1). For all of the remaining parameters, no significant difference was identified between the groups (P > 0.05).

**Table 1.** Baseline characteristics of study patients with thrombus localization in pulmonary artery (n = 122)

Main	Proximal (n = 80)		Distal (n = 42)	
	Branch	Segmental	Subsegmental	P
Variables	(n = 9)	(n = 71)	(n = 34)	(n = 8)
Mean age (years)	70 ± 10	63 ± 12	63 ± 15	66 ± 11	0.430
Women, n (%)	3 (33%)	43 (61%)	14 (41%)	3 (38%)	0.507
Admission symptoms, n (%)	0.926
Dyspnea	6 (67%)	47 (66%)	24 (71%)	6(75%)	0.941
Angina pectoris	1 (11%)	22 (31%)	10 (29%)	2 (25%)	0.658
Hemoptysis	1 (11%)	5 (7%)	2 (6%)	0 (0%)	0.821
Syncope	1 (11%)	5 (7%)	1 (13%)	1 (12.5%)	0.678
Symptom duration, n (%)	0.308
< 6 hours	2 (22%)	3 (4%)	2 (6%)	0 (0%)	0.150
6-12 hours	1 (11%)	4 (6%)	2 (6%)	1 (12.5%)	0.828
12-24 hours	3 (33%)	17 (24%)	4 (12%)	1 (12.5%)	0.343
> 24 hours	3 (33%)	47 (66%)	26 (77%)	6 (75%)	0.099
Hypertension, n (%)	5 (56%)	31 (44%)	11 (32%)	2 (25%)	0.411
Diabetes mellitus, n (%)	2 (22%)	17 (24%)	4 (12%)	3 (38%)	0.335
Coronary artery disease, n (%)	2 (22%)	14 (20%)	8 (24%)	3 (38%)	0.711
Chronic obstructive pulmonary disease, n (%)	3 (33%)	13 (18%)	4 (12%)	0 (0%)	0.243
Immobilization, n (%)	3 (33%)	14 (20%)	7 (21%)	1 (12.5%)	0.741
Previous pulmonary embolism, n (%)	0 (0%)	6 (9%)	2 (6%)	0 (0%)	0.652
Previous deep venous thrombosis, n (%)	0 (0%)	5 (7%)	2 (6%)	0 (0%)	0.739
Previous history of surgery, n (%)	2 (22%)	14 (20%)	10 (29%)	0 (0%)	0.307
Thrombolytic usage, n (%)	2 (22%)	6 (9%)	0 (0%)	0 (0%)	0.073
Cardiopulmonary resuscitation requirement, n (%)	4 (44%)	11 (16%)	2 (6%)	0 (0%)	0.016
Early mortality, n (%)	4 (44%)	12 (17%)	1 (3%)	0 (0%)	0.007
Hemodynamic findings
Heart rate (beats/minute)	123 ± 21	112 ± 19	104 ± 18	98 ± 8	0.023
Systolic blood pressure (mmHg)	97 ± 21	106 ± 20	108 ± 13	112 ± 16	0.494
Diastolic blood pressure (mmHg)	59 ± 20	64 ± 16	69 ± 10	65 ± 9	0.476
Oxygen saturation (%)	77 ± 10	85 ± 8	89 ± 9	87 ± 14	0.004
Electrocardiography parameters, n (%)					
Atrial fibrillation	3 (33%)	16 (23%)	6 (18%)	1 (12.5%)	0.687
Right bundle branch block	4 (44%)	20 (28%)	8 (24%)	1 (12.5%)	0.476
S1Q3T3	3 (33%)	15 (21%)	6 (18%)	0 (0%)	0.361
T wave changes	5 (56%)	29 (41%)	7 (21%)	3 (38%)	0.126
Echocardiography parameters					
Left ventricle ejection fraction (%)	54 ± 10	54 ± 10	56 ± 8	49 ± 15	0.694
Right ventricular dilatation, hypokinesia, n (%)	8 (100%)	43 (83%)	14 (52%)	4 (57%)	0.006
Severe tricuspid regurgitation, n (%)	3 (38%)	14 (26%)	5 (17%)	0 (0%)	0.232
Systolic pulmonary artery pressure (mmHg)	53 ± 10	53 ± 21	42 ± 15	32 ± 12	0.004
Laboratory findings					
Gamma-glutamyl transferase (IU/l)	86 (49-117)	58 (36-95)	35 (22-55)	24 (17-36)	< 0.001
Alkaline phosphatase (IU/l)	102 (78-156)	97 (74-117)	83 (62-103)	70 (62-81)	0.231
Hemoglobin (g/dl)	12.7 ± 2	12.8 ± 1.9	13.4 ± 2.2	13.3 ± 1.6	0.528
Presence of anemia	5 (56%)	25 (35%)	12 (35%)	2 (25%)	0.585
Creatinine (mg/dl)	1.35 (0.9-2.1)	1.0 (0.8-2.3)	0.96 (0.8-1.2)	1.1 (0.7-1.3)	0.272
Troponin I (ng/ml)	0.08 (0.03-0.26)	0.05 (0.03-0.09)	0.25 (0.01-0.57)	0.12 (0.01-0.6)	0.056
Alanine aminotransferase (IU/l)	38 (18-108)	31 (17-58)	20 (15-48)	24 (21-157)	0.268
D-dimer >1500 ng/ml	8 (89%)	45 (71%)	19 (70%)	5 (63%)	0.642

GGT levels were found to be correlated with admission heart rate (r = 0.398; P < 0.001), systolic blood pressure (r = -0.251; P: 0.018), diastolic blood pressure (r = -0.316; P = 0.003), oxygen saturation (r = -0.454; P < 0.001), presence of right bundle branch block (r = 0.217; P = 0.009), alkaline phosphatase level (r = 0.528; P < 0.001), alanine aminotransferase level (r = 0.508; P < 0.001) and creatinine level (r = 0.256; P = 0.005). There was no significant correlation between GGT levels and any other laboratory findings (P > 0.05).

The results from the univariate and multivariate logistic regression analyses on proximal pulmonary artery involvement are shown in Table 2. GGT levels on admission, heart rate, oxygen saturation, right ventricular dilatation/hypokinesia, pulmonary artery systolic pressure and cardiopulmonary resuscitation requirement were found to have prognostic significance in the univariate analysis. The multivariate logistic regression model using a forward stepwise method showed that GGT levels on admission (odds ratio = 1.044; 95% confidence interval: 1.011-1.079; P = 0.009) and pulmonary artery systolic pressure (odds ratio = 1.063; 95% confidence interval = 1.005-1.124; P = 0.033) remained associated with proximal pulmonary artery involvement after adjusting for the significant variables in the univariate analysis, and were correlated with the GGT level.

**Table 2:**
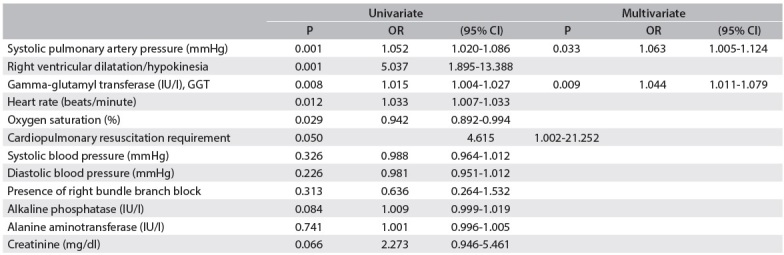
Univariate and multivariate analyses on proximal pulmonary artery involvement

All the variables from Table 1 were examined and only those significant at the level P < 0.05 and correlated with GGT level are shown in the univariate analysis. The multivariate logistic regression model with forward stepwise method included all univariate predictors and those with correlated GGT level. CI = confidence interval; OR = odds ratio.

**Figure 2:**
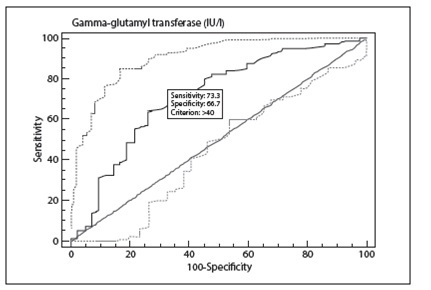
Receiver operating characteristic (ROC) curve for gamma-glutamyl transferase (GGT) levels to predict proximal pulmonary artery involvement.

According to the ROC curve analysis, the optimal cutoff value for GGT for predicting proximal pulmonary artery involvement was measured as > 40 IU/l, with 73.7% sensitivity, 66.7% specificity, 80.8% positive predictive value and 57.1% negative predictive value (AUC 0.851; 95% confidence interval 0.777-0.908; Figure 2). Furthermore, an admission GGT level of > 139 IU/l [n = 8 (7%)] had specificity of 95%, sensitivity of 7.5%, positive predictive value of 75% and negative predictive value of 35%. In contrast, an admission GGT level < 20 IU/l [n = 13 (11%)] had sensitivity of 95%, specificity of 28.6%, positive predictive value of 72% and negative predictive value of 75%. 

## DISCUSSION

To the best of our knowledge, this is the first study to demonstrate the correlation between GGT levels and the degree of pulmonary artery involvement. The findings revealed that there was a significant correlation between increased existing embolism load in the pulmonary artery and increased serum GGT levels. This finding may prove to be useful both in treatment planning and as a predictor of mortality. 

Pulmonary embolism is a cardiovascular disease with a high potential for mortality that can progress into a broad spectrum of clinical manifestations (ranging from silent clinical progress to hemodynamic instability). Although there have been studies indicating a direct correlation between the obstruction level of the pulmonary artery, coagulation load and mortality,^5^ Mansencal et al. did not find any discriminant correlation between pulmonary hemodynamic data and prognosis.^19^ Similarly, Araos et al. demonstrated that coagulation load was a weak predictor of mortality,^20^ while Ghuysen et al. stated that pulmonary arterial systolic pressure and the obstruction index of the pulmonary artery were correlated with clinical severity, but were unsuitable for use as mortality predictors.^21^ In risk classification, detection of proximal extension in pulmonary embolism patients is a prognostic factor with an unrecognized value.^5^

The cardiac biomarkers troponin and natriuretic peptide have emerged as promising tools for evaluating the risk among patients diagnosed with APE. Increased cardiac troponin levels in APE can be explained by the increased right ventricular wall pressure, which leads to compression of the right coronary artery and direct myocardial damage.^22^ Moreover, the risks of mortality and complications have been reported to be higher among patients with elevated cardiac troponin-T during the acute phase of pulmonary embolism.^6^ Natriuretic peptide is used both as a diagnostic and as a prognostic marker for patients suffering from congestive heart failure, since it is a prohormone that does not accumulate to any significant degree in normal ventricular myocytes. In this regard, its levels increase significantly a few hours after acute myocardial contractions.^23^ Similar to cardiac troponins, brain natriuretic peptide and N-terminal pro-brain natriuretic peptide are associated with right ventricle dysfunction in APE.^24^ ^25^

GGT assaying is a liver function test that is used as a sensitive indicator of alcohol consumption and hepatobiliary dysfunction.^26^ GGT is found not only in the liver, but also in the kidneys and vascular epithelium, as well as in the extracellular fluid, where it is attached to albumin carrier molecules and lipoproteins.^27^ Previous studies have shown that increased GGT levels are associated with decreased oxygen levels and increased levels of inflammatory markers.^28^ ^29^ Moreover, the CARDIA study has shown that increased GGT levels are associated with oxidative stress, inflammatory events and diet.^28^ Wanamethee et al. identified a correlation between high GGT levels and cardiovascular diseases and stroke in a prospective study on 6,997 patients.^12^ Furthermore, different studies have indicated that serum GGT levels can be used as an early predictor during the development of cardiovascular events and metabolic syndrome.^30^ ^31^

APE is a complex process involving different mechanisms, and GGT levels increase as a result of contributions from all these mechanisms. In the event of a pulmonary artery embolism, embolism severity and involvement levels increase directly proportionally to the coagulation load. By extension, hypoxia intensifies, and increased activity is seen in the neurohormonal and adrenergic systems. In addition, an increased coagulation load leads to an increase in pulmonary arterial pressure, and thus an increase in hepatic congestion, and the increase in coagulation load is further correlated with an increase in GGT levels. As shown in the current study, GGT levels increase with increasing numbers of pulmonary artery segments involved, because of the activity of the mechanisms described above. Moreover, Zorlu et al. showed that increased GGT levels could be a predictor of early mortality in cases of APE.^14^

Our study has some limitations. The most important of these is the single-center nature of the findings. B-type natriuretic peptides are established biomarkers for pulmonary embolism, but this was not examined in our study. Selection bias, and the likelihood that serious cases may have been lost before any tests or evaluations could be conducted, even before admittance to the hospital, may also have influenced this result, in that patients with more severe proximal involvement are more likely to die acutely. Furthermore, this study was conducted in a tertiary care center: patients with more severe complaints are referred to such centers, while those with milder symptoms are not brought to tertiary care centers, but are managed in secondary care hospitals. 

## CONCLUSION

The present study identified a significant correlation between serum GGT levels and pulmonary artery involvement levels, i.e. the severity of an embolism event.
